# Cancer/testis antigens expression during cultivation of melanoma and soft tissue sarcoma cells

**DOI:** 10.1186/s13569-020-0125-2

**Published:** 2020-02-04

**Authors:** Anna Danilova, Vsevolod Misyurin, Aleksei Novik, Dmitry Girdyuk, Natalia Avdonkina, Tatiana Nekhaeva, Natalia Emelyanova, Nino Pipia, Andrey Misyurin, Irina Baldueva

**Affiliations:** 1 N.N. Petrov’ National Medical Cancer Research Center, Leningradskaya str., 68, Pesochniy, Saint-Petersburg, 197758 Russian Federation; 2 N.N. Blokhin’ National Medical Cancer Research Center, Kashirskoye sh. 24, Moscow, 115478 Russian Federation; 3 Department of Oncoimmunology, N.N. Petrov’ National Medical Cancer Research Center, Leningradskaya str., 68, Pesochniy, Saint-Petersburg, 197758 Russian Federation

**Keywords:** Cancer/testis antigens, Soft and bone tissues sarcoma, Melanoma, Tumor cells lines, Dendritic cell vaccine

## Abstract

**Background:**

Autologous dendritic cells (DC) loaded with tumor-associated antigens (TAAs) are a promising approach for anticancer immunotherapy. Polyantigen lysates appear to be an excellent source of TAAs for loading onto the patient’s dendritic cells. Cancer/testis antigens (CTA) are expressed by a wide range of tumors, but are minimally expressed on normal tissues, and could serve as a universal target for immunotherapy. However, CTA expression levels can vary significantly in patients with the same tumor type. We proposed that patients who do not respond to DC-based therapy may have distinct features of the CTA expression profile on tumor cells.

**Patients and methods:**

We compared the gene expression of the principal families CTA in 22 melanoma and 27 soft tissue and bone sarcomas cell lines (STBS), received from patients and used for DC vaccine preparation.

**Results:**

The majority (47 of 49, 95.9%) cell lines showed CTA gene activity. The incidence of gene expression of *GAGE*, *NYESO1*, *MAGEA1*, *PRAME*’s was significantly different (adj. p < 0.05) between melanoma and sarcoma cell lines. The expression of the *SCP1* gene was detected neither in melanoma cells nor in the STBS cells. Clustering by the gene expression profile revealed four different expression patterns. We found three main patterns types: hyperexpression of multiple CTA, hyperexpression of one CTA with almost no expression of others, and no expression of CTA. All clusters types exist in melanoma and sarcoma cell lines. We observed dependence of killing efficacy from the *PRAME* (rho = 0.940, adj. p < 0.01) expression during real-time monitoring with the xCELLigence system of the interaction between melanoma or sarcoma cells with the T-lymphocytes activated by the lysate of selected allogenous melanoma cell lines with high expression of CTA.

**Conclusion:**

Our results demonstrate that one can use lysates from allogeneic melanoma cell lines as a source of CTA for DC load during the production of anticancer vaccines for the STBS treatment. Patterns of CTA expression should be evaluated as biomarkers of response in prospective clinical trials.

## Background

Conventional treatment (i.e., surgery, chemotherapy, radiotherapy) of disseminated malignant tumors has significant limitations since it is not effective in many cases. In recent decades, several immunotherapy methods became the breakthrough in oncology. Among them—recombinant cytokines, immune checkpoint inhibitors T-cell-mediated adaptive therapies, and dendritic cells (DC)-based vaccines [1].

Autologous DCs loaded with tumor-associated antigens (TAA) are a promising tool for anticancer therapy. They stimulate an integral antitumor response and contribute to the formation of immunological memory [2]. DC-based vaccine therapy is a safe approach capable of forming long-term protective immunity [3]. Tumor lysates can be used as an excellent source of TAA for dendritic cells. DC pulsed with tumor lysate can induce an immune response through the generation of cytotoxic T-lymphocytes specifically activated against tumor antigens, which leads to tumor regression in animal models [4]. DCs, activated by whole or lysed autologous and allogeneic tumor cells but not mRNA isolated from tumor cells, produce the most effective immunological response [5, 6]. One of the first vaccines was developed based on autologous DC derived from peripheral blood monocytes and lysate-activated allogeneic cutaneous melanoma and prostate cancer cells [7]. This approach showed the formation of delayed-type tumor-specific hypersensitivity reactions, immunological, and clinical responses that leads to the increased survival rate in patients with disseminated forms of the disease [8–10]. As many as 204 clinical studies with DC vaccines are registered at ClinicalTrial.gov portal by Jan 2019. One can found trials of clinical and immunological efficacy of vaccines based on DC with tumor lysate (191 tumor lysate-pulsed dendritic cell), 2 with whole tumor cells, 11 based on hybrids of DC and tumor cells (dendritic cell/tumor fusion vaccine) among them [11].

Despite promising results, many patients remain refractory to DC-based approaches. On the one hand, this phenomenon may be related either to the lack of sufficient immunological hazard signals or to the absence of adequate immunogenic danger signals in the process of differentiation monocytes in DC in vitro and the violation of processing and presentation of antigens by vaccine DC. On the other hand, this could be related to the difference in the antigenic profile of tumor cells used for the preparation of lysates and the patient’s tumor cells. Cancer/testicular antigens (CTA) are the preferred targets among TTA since they can be hyper expressed in tumors but not in normal tissue with the exemption of testicle and placenta, which are immune-privileged organs. They are highly immunogenic because the immune system of the adult organism “does not know” CTAs and, and thus are not tolerated [12, 13]. CTA can be considered as a universal target for immunotherapy since a wide range of tumors expresses them. However, the level and profile of CTA expression may be significantly different for patients with the same diagnosis [14–18].

So far, there are over 270 distinguished CTAs in CT database (http://www.cta.lncc.br). CTAs are composed of gene families of closely associated members. They are generally characterized into two groups: CT-X antigens that are located on the X chromosome and non-X CTAs, that are encoded by autosomes and the Y chromosome.

Some antigens, including MAGE antigens, are represented by multi-antigen families of related antigens. Some families consist of only one member (i.e., SCP1) [40]. Each gene in the gene family is located in the same chromosome locus and is governed by the same enhancer. So, members of one family are almost always coexpressed with the same level of expression. Moreover, there is high gemology of gene family members in the structure and function. They also share immunogenic properties. We hypothesized that it can be enough to determine the activity of only one gene from each family to assess the immunogenic profile of the tumor. We selected 11 genes with the most well-known expression in neoplasms on the one hand and maximal structure differences on the other. We assumed that the determination of the CTA expression profile GAGE, HAGE, NY-ESO1, MAGEA1, PASD1, SCP1, SEMG1, SLLP1, SPANXA1, SSX1, and PRAME will allow characterizing in sufficient detail the immunophenotype of the tumor cells that can be used for the preparation of lysate during DC vaccine production. It was already noted that tumors of different histotype may share a similar antigenic profile. Based on this data we proposed that DC vaccines loaded with melanoma cells could be used for the treatment of patients with other types of cancer in case their tumor cells express similar antigens [19–21].

Treatment of soft tissue and bone sarcomas (STBS) is an unmet medical need. The prognosis of soft tissue sarcoma remains poor in the recurrent and metastatic setting. Substantial genetic and histologic heterogeneity of this group makes treatment development very complicated. Nevertheless, STBS are immunogenic because their cells express the broad spectrum of CTA [22–24]. The universality of immunologic approaches and possible immunogenic targets in STBS promotes the development of immunotherapeutic methods for their therapy. The cultivation of tumor cells allows obtaining unlimited amounts of cellular material with the desired properties for the creation of antitumor vaccines. STBS cells are delicate in cultivation and possess low proliferative activity in vitro at the same time [25]. This is why it could be preferable to use cultured melanoma cells rich in CTA to load and activate DC in patients with STBS. A mandatory condition for the creation of an effective DC vaccine, in this case, is the similarity of the CTA expression profiles between the cells used for the loading and activation of DC and the patient’s tumor cells.

We conducted a comparative study of gene expression profiles of the main CTA types of cultured melanoma cells and STBS cells derived from patients and estimate the efficacy of this therapy in the experimental cell models with cytotoxic T-lymphocytes, specifically activated by mature DC loaded with melanoma-derived cell lysate, and with sarcoma cells derived from tumors of patients with different antigenic profiles.

## Materials and methods

### Tumor cell cultures

Fresh tumor samples (22—melanoma and 27—STBS) were obtained from 49 patients receiving surgical treatment at the N.N. Petrov National Medical Research Center of Oncology from 2003 to 2018. The material for this study was collected after receiving the patients’ informed consent. Cell cultures were established in all cases. Histological subtypes of tumors and their localization are shown in Table 1. All tumors were high grade with the exemption of four myxoid liposarcomas with the low grade. Primary tumors were the source of the samples in 16.4% of cases (8/49). The tumor samples were disaggregated mechanically in the Medimachine (Agilent Technologies, USA) and placed in growth medium DMEM/F12 of the following composition: 20% heat-inactivated fetal bovine serum (Gibco, USA), 365 mg/l glutamine, 5 µg/ml insulin, 5 µg/ml transferrin, 5 ng/ml selenium (Invitrogen), 100 U/ml penicillin, 100 µg/ml streptomycin (Sigma-Aldrich, USA). The cell suspensions were directly distributed into 25 cm^3^ culture flasks (BD Bioscience, USA) at 37 °C in a humidified atmosphere of 5% CO_2_ according to the Freshney method [26]. When cells reached sub-confluence, they were dispersed with 0.25% trypsin–0.02% EDTA (Sigma-Aldrich, USA) and seeded in a new culture plate. Fibroblast growth-inhibitory cocktail Human FibrOut™ 9 (Chi Scientific Inc., USA) was used for fibroblast growth prevention. Cells have been cultured continuously for at least ten passages. CTA expression was tested at the 10th passage.

**Table 1:** Histological subtypes and localization of skin melanoma and soft tissue and bone sarcomas used as a source of cell lines

Tumor	Primary	Recurrence	Metastatic		Total
Melanoma					
Melanotic					
Spindle cell	1	0	0	–	1
Epithelioid cell	2	0	10	Soft tissue (3) Lymph nodes (4) Lung (2) Thyroid (1)	12
Amelanotic					
Spindle cell	0	0	1	Soft tissue (1)	1
Epithelioid cell	0	2	6	Soft tissue (2) Lymph nodes (4)	8
STBS					
Osteosarcoma	1	0	4	Lung (4)	5
Liposarcoma					
Myxoid	1	1	4	Lung (1) Lymph nodes (1) Breast (1) Extraperitoneal (1)	6
Pleomorphic	0	0	1	Lung (1)	1
Synovial sarcoma	0	2	2	Lung (1) Pleural cavity (1)	4
Myxofibrosarcoma	1	1	2	Lung (1) Soft tissue (1)	4
Leiomyosarcoma	0	0	1	Lung (1)	1
Rabdomyosarcoma	0	1	1	Lung (1)	2
Alveolar sarcoma	0	0	1	Lung (1)	1
Clear cell sarcoma	0	1	0	–	1
Chondrosarcoma	1	0	0	–	1
Dermatofibrosarcoma	1	0	0	–	1
Total	8	8	33		49

Numeric in brackets represents the number of cases

### RNA extraction and reverse transcription-polymerase chain reaction (RT-PCR)

Total RNA has been extracted from cells accordingly using RNA-extract kit (Genetechnology, Russia) to evaluate the expression of individual CTA genes. Reverse transcription reactions were performed using an enzyme and RevertAid Reverse reagent kit (Fermentas, USA) in conditions suggested by the manufacturer. Two microgram of RNA was used to perform the reaction of reverse transcription. For the annealing, a mixture of six-membered random primers (Synthol, Russia) was used. A working mixture without the addition of RNA was used as a negative control; the sample was adjusted to the final volume by deionized water. After DNA synthesis, cancer/testis gene expression was quantitated and normalized to ABL expression. Relative cancer-testis genes expression levels were calculated using the LightCycler (Roshe) software. We used the following primers and probes:

ABL primers 5′-CGTTGCACTGTATGATTTTGTGGC-3′; 5′-GCTTCACACCATTCCCCATTGTG-3′; probe R6G-AGCATAA(C-LNA)TAAAGGT(G-LNA)AAAAG(C-LNA)TCC-BHQ1.

GAGE primers 5′-AGCTGCTCAGGAGGGAGAGGAT-3′; 5′-GGTGACCCTGTTCCTGGCTA-3′; probe 5′-(R6G)-CATCTGCAGGTCAAGGGCCGAAGCCTGAA-(BHQ1)-3′; HAGE primers 5′-GCCACAAGTGCCATGTCAAA-3′; 5′-CCTTCAAGTCATCCCACGTT-3′; probe 5′-(R6G)-AGCAGATAGTTGGAGGAAAGAAAATTTTAA-(BHQ1)-3; MAGEA1 primers 5′-GAAGGAACCTGACCCAGGCT-3′; 5′-AATCCTGTCCTCTGGGTTGG-3′; probe 5′-(R6G)-TGTGAGGAGGCAAGGTTTTCAGGGGAC-(BHQ1)-3′; NY-ESO-1 primers 5′-TCTGAAGGAGTTCACTGTGT-3′; 5′-AGACAGGAGCTGATGGAGAG-3′; probe 5′-(R6G)-AACATACTGACTATCCGACTGACTGCTGCA-(BHQ1)-3′; PASD1 primers 5′-GTGGGAAATGTTTGCATTCT-3′; 5′-AGCTTCATCACTGACTGCCT-3′; probe 5′-(R6G)-TCAGCTCCTGCAGCAACTTTACACTTC-(BHQ1)-3′; SCP1 primers 5′-AAAAGGAACAGAACAAGAAC-3′; 5′-TGTGGTAATGGCAGTTAACT-3′; probe 5′-(R6G)-CCAAGCCAGAGAGAAAGAAGTACATGATTT-(BHQ1)-3′; SEMG1 primers 5′-TCCTCATCTTGGAGAAGCAA-3′; 5′-TGGGAAAATTCACTTGGTAA-3′; probe 5′-(R6G)-ATGGGACAAAAAGGTGGATCAAAAGGCC-(BHQ1)-3′; SLLP1 primers 5′-ACTTCGGGCTGGACGGATAC-3′; 5′-GCGTTGAAACCGCTTGTGAA-3′; probe 5′-(R6G)-ATACAGCCTGGCTGACTGGGTCTGCCTTGCTTA-(BHQ1)-3′; SPANXA1 primers 5′-GAGGAGCGTCCCCTGTGATT-3′; 5′-AGCAGGTTGCGGGTCTGAGT-3′; probe 5′-(R6G)-AGGCCAACGAGATGATGCCGGAGACCCCAA-(BHQ1)-3′; SSX1 primers 5′-GTATATGAAGAGAAACTATAAGG-3′; 5′-TATTACACATGAAAGGTGGG-3′; probe 5′-(R6G)-ATGACTAAACTAGGTTTCAAAGTCACC-(BHQ1)-3′; PRAME primers 5′-TCTTCCTACATTTCCCCGGA-3′; 5′-GCACTGCAGACTGAGGAACTGA-3′; probe 5′-(FAM)AAGGAAGAGCAGTATATCGCCCAGTTCACC-(TAMRA)-3′. The primers have been produced by Evrogen (Russia). The synthesis of fluorescent probes was carried out by DNA-synthesis (Russia). Relative expression is reported (ΔCt and ΔΔCt calculations). Besides, a more sensitive qPCR assay was used, in which cancer/testis gene RNA copy numbers in samples were extrapolated from known copy numbers of the serially diluted plasmid with cloned cDNA of cancer/testis gene and normalized to B2M expression. All samples were run in duplicates. The reaction has been performed on a Light Cycler 96 PCR amplifier (Roshe, Switzerland).

Considering the exponential nature of the data obtained by qPCR, CTA expression levels were standardized with log1p function with logarithm’s base equals 2 (log_2_(x + 1)). Further, by an expression we mean precisely its transformed values.

### Preparation of antigen-specific T cell cultures

#### Tumor lysate preparation

Four melanoma cell cultures were selected for tumor lysate preparation by their CTA expression profile. Cells were removed from the substrate, evaluated for viability, and mixed in equal proportions. Six consecutive cycles of instant freezing to − 196 °C and thawing to room temperature in phosphate-buffered saline without cryoprotectant were performed. The quality of the lysis was monitored with a 0.1% trypan blue stain and assessed with a light microscope. Then, centrifugation for 10 min at 200*g* was carried out, followed by filtration of the super-sedimentary fraction through a 0.2 µm filter and packing of tumor lysate into cryovials with storage at − 20 °C before use.

#### Dendritic cell culture

Mononuclear cells from the peripheral blood of patients were extracted by centrifugation in a density gradient “Ficoll-Paque Premium” GE Healthcare “ (Great Britain) by Boyum method [27]. Monocytes (CD14^+^) and lymphocytes (CD3^+^) were separated by plastic adhesion [28]. Monocytes were cultured in a serum-free medium CellGro DC, in the presence of 72 ng/ml GM-CSF and 15 ng/ml IL-4 (CellGenix, Germany), which were added in the first, third and fifths days of cultivation. On the seventh day of cultivation for the maturation of DC, tumor antigens were introduced, based on the ratio of 1 DC/3 lysed tumor cells, growth factors—GM-SCF (72 ng/ml), IL-4 (15 ng/ml) (CellGenix, Germany) and TNF-α (20 ng/ml) (BD Bioscience, USA). DCs were collected after 48 h.

#### T-cell culture

We have used a method described by Märten et al. [29] with modifications. The fraction of autologous lymphocytes were cocultured with mature DCs in the presence of 72 ng/ml GM-CSF, 15 ng/ml IL-4, (CellGenix, Germany), 50 IU/ml IL-2, 10 ng/ml IL-7 and 20 ng/ml TNF-α (BD Bioscience, USA) for 7 days, adding cytokines every 48 h. The procedure was repeated twice. Antigen-specific T-cells were thus specifically activated and expanded in culture. The specificity of cells activation was confirmed in ELISpot tests.

### Analysis and sorting of CD8^+^ T cells

The extraction of specifically activated CD8^+^ T-cells after their cocultivation with antigen-loaded DCs were carried out via the negative magnetic separation method, using the EasySep Magnet device and were isolated from cell suspension using the EasySep Human CD8^+^ T Cell EnrichmentKit (STEMCELL Technologies Inc., Canada).

CD8^+^ T lymphocytes suspension was analyzed by flow cytometry. Flow cytometric measurements were performed on a FACSCanto II cytometer and analyzed using BD FACS Diva Version 8.0.1 (BD Bioscience, USA). These cells were predominantly CD3^+^CD8^+^HLA-DR^+^ T-lymphocytes producing Granzyme B, Perforin, INFγ.

Produced activated CTL were used for real-time cytotoxicity assay.

### Real-time cytotoxicity assay (xCELLigence)

Tumor cells had been sown previously in an amount of 2 × 10^4^ per well in E-16 Plates (ACEA Bioscience., USA) in order to evaluate the efficacy of the interaction of activated CD8^+^ T-lymphocytes with tumor cells in the cell analyzer xCELLigence (ACEA Bioscience., USA). A 50-µl medium was added to plates for the measurement of background values. Consistently, target cells were seed in an additional 100 µl medium at a density of 20,000 cells per well. The plates were left in CO_2_ incubator conditions for 30 min to minimize turbulent fluid flows. Activated CTL were then introduced into the system at a ratio of 1 tumor cell/5, 10, 50 lymphocytes to determine their optimal amount. Melanoma cells used as target cells, from which cell lysates were prepared for loading and activation of DCs at the first step. STBS cells with CTAs were used as target cells in the second step. The plates were placed in the device. Electrical signals were recorded over a period of 48 h. Changes in electrical impedance were expressed as a dimensionless cell index (CI) value, which was derived from relative impedance changes corresponding to cellular coverage of the electrode sensors, normalized to baseline impedance values with medium only. Cell index values were recorded every 5 s during the first hour, and then every 15 s, until the end of the experiment, which lasted 48 h in total. Thus, based on the STBS cells proliferation on the E-plate, with or without CTLs, we could determine the cytotoxic effects of this therapy on target cells. Only HLA-A2^+^ cells were used in the experiments. The percentage of cell lysis in the process of interaction of T-lymphocytes and STBS cells was calculated using the method described earlier [30].

### Statistical analysis

We used statistical notation according to Everitt and Pickles [31]. For exploratory data analysis, we applied descriptive statistics such as median, 25th and 75th percentile, min, max. We also applied the nonparametric U-Mann–Whitney test; Spearman’s correlation coefficient, exact Fisher’s criteria, complete-linkage hierarchical cluster analysis by with Euclidian distance. For determining the relevant number of clusters, we used R’s “NbClust” package [32], which contains 28 indices and two graphical methods for assessing it by using the majority rule. We used R v.3.5.1 for conducting the statistical tests and visualization of the results. The adjustments of p-values for multiple comparisons were performed with the Benjamini–Hochberg procedure. Adjusted p-values less than 0.05 were considered statistically significant.

## Results

### Tumor cell cultures

**Fig. 1:**
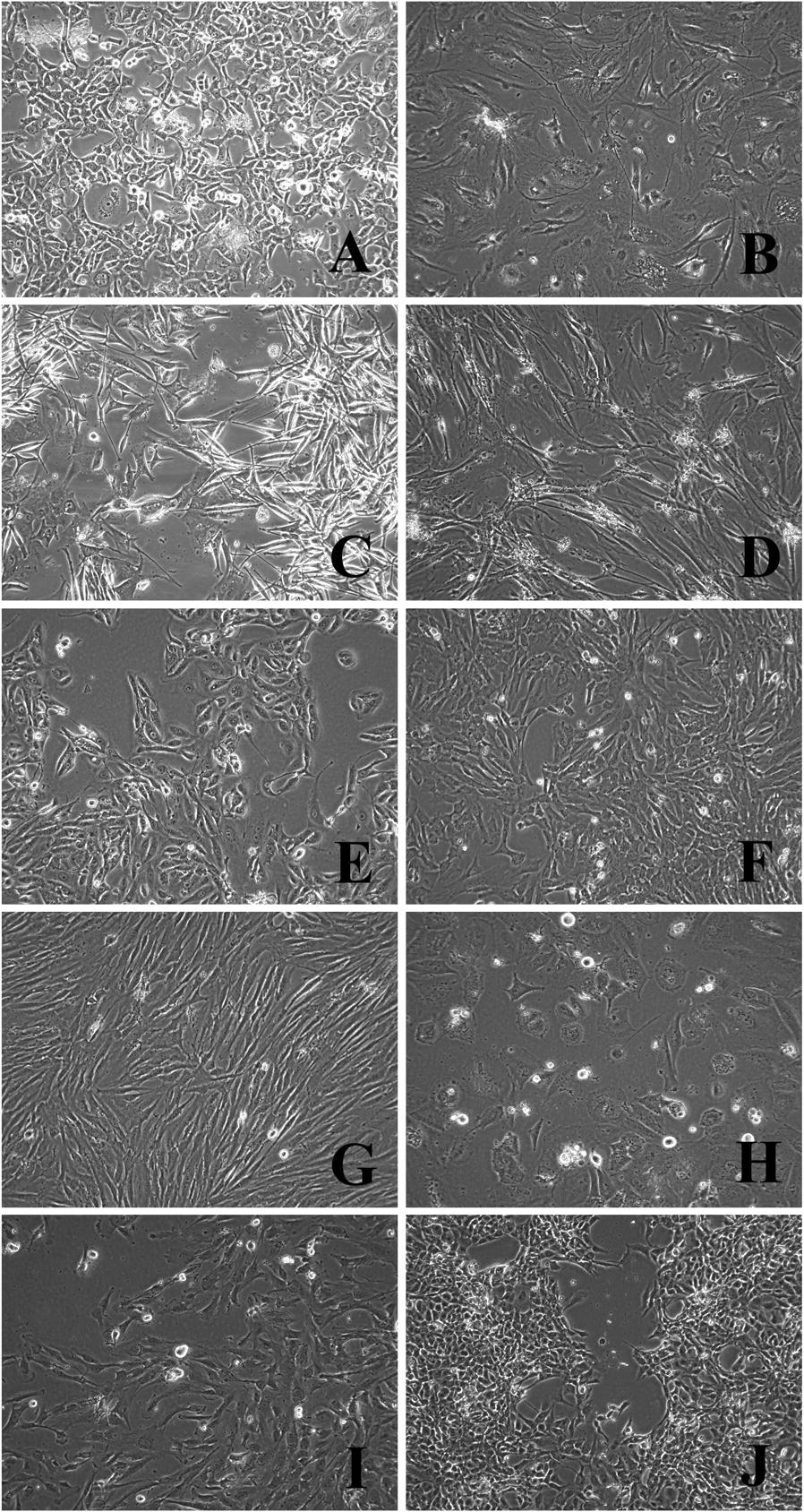
Morphology of the established melanoma cell cultures and STBS cell cultures: **A** # 226 melanoma, 25 passage; **B** # 694 melanoma, 10 passage; **C** # 716 synovial sarcoma, 25 passage; **D** # 862 rhabdomyosarcoma, 13 passage; **E** # 702 liposarcoma, 22 пaccaж; **F** # 927 alveolar sarcoma, 10 passage; **G** # 921 osteosarcoma, 10 passage; **H** # 699 leiomyosarcoma, 16 passage; **I** # 925 chondrosarcoma, 11 passage; **J** # 678 myxofibrosarcoma, 16 passage. Inverted microscope, phase contrast, ×100

All extracted tumor cell cultures were characterized by high morphological heterogeneity within one histological type. Melanoma cultures demonstrated the most pronounced variety of morphological types, including fibroblast-like, fusiform, epithelioid, stellate, polygonal, and round shape of cells (Fig. 1A, B). The variability of STBS cell culture morphology correlated with a variety of histological types: chondro-, rhabdo- and leiomyosarcoma cultures were represented mainly by fibroblast-like strongly elongated fusiform cells (Fig. 1D, H, I). In liposarcoma cultures, smaller polygonal cells (Fig. 1e) were observed. In synovial sarcoma cultures, both strongly elongated cells and polygonal process form could be detected (Fig. 1C). Myxofibrosarcoma had cells with a strongly fibrous cytoplasm of the striated structure were represented by fibroblast-like cells (Fig. 1J). Alveolar sarcoma in the culture was described by small stellate cells (Fig. 1F). Osteosarcoma in the culture had mainly large rounded and polygonal cells (Fig. 1G). Cells of at least ten passages were cultured, after which their antigenic properties were studied.

### CTA expression

The activity of CTA genes was registered in 47 of 49 (95.9%) samples of melanoma and STBS cell lines. Two cultures of liposarcoma—S944.1 and S945—did not express any of the studied genes. A more pronounced CTA expression profile marked melanoma cells compared to the STBS group. All cultures were characterized by high heterogeneity of studied genes expression (Table 2). The expression of the *SCP1* gene was detected neither in melanoma cells nor in the STBS cells. There was no expression of *SEMG1* and *SPANXA1* in STBS cells. The activity of the rest of the studied genes varied substantially even within one histological tumor type, especially in the STBS group. Among STBS, myxofibrosarcoma (8 genes out of 11), osteosarcoma (7/11), dermatofibrosarcoma (7/11), and synovial sarcoma (6/11) cells demonstrated the most significant expression of CTA genes. In cell samples of chondrosarcoma and clear cell sarcoma, gene expression was minimal (3/11). We excluded 4 CTA (*HAGE*, *SCP1*, *SEMG1*, *SPANXA1*) before statistical analysis due to a slight or complete lack of their expression (genes expressed in 6, 0, 3, 2 cell lines respectively).

**Table 2:** Analysis of cancer/testicular genes expression in melanoma and STBS cells

Quantitative CTA expression level, median, Q1–Q3, min–max			
Gene	Melanoma cells	Soft tissues and bones sarcoma cells	U-Mann–Whitney test, p-value (adjusted p-value)
*GAGE*	2.05 0–0.73–3.33–6.24	0 0–0–1.20–3.94	< *0.001* (*<0.001*)
*HAGE*	0 0–0–0–0.07	0 0–0–0–2.37	–
*NY*-*ESO1*	2.97 0–1.20–3.93–7.36	0 0–0–1.37–5.13	< *0.001* (*0.003*)
*MAGEA1*	1.86 0–0.69–3.27–4.49	0 0–0–0.61–4.06	< *0.001* (< *0.001*)
*PASD1*	0.60 0–0.05–1.59–2.85	0 0–0–0.01–2.78	*0.002* (*0.013*)
*SCP1*	0 0–0–0–0	0 0–0–0–0	–
*SEMG1*	0 0–0–0–1.18	0 0–0–0–0	–
*SLLP1*	0.02 0–0–0.09–2.69	0 0–0–0.01–1.07	0.049 (0.113)
*SPANXA1*	0 0–0–0–1.06	0 0–0–0–0	–
*SSX1*	0 0–0–0.01–4.50	0 0–0–0.01–9.31	0.837 (0.887)
*PRAME*	4.86 2.27–4.06–5.53–6.40	0.01 0–0–2.11–2.73	< *0.001* (<* 0.001*)

The incidence of gene expression of the CTA				
Gene	Positive cases/total (positive %)		Fisher’ exact test, p-value (adjusted p-value)	Odds ratio (STBS/melanoma)
	Melanoma cells	Soft tissues and bones sarcoma cells		
*GAGE*	21/22 (95.5%)	10/27 (37.04%)	< *0.001* (<* 0.001*)	0.028
*HAGE*	1/22 (4.54%)	5/27 (18.52%)	–	4.773
*NY*-*ESO1*	21/22 (95.54%)	15/27 (55.56%)	*0.003* (*0.014*)	0.060
*MAGEA1*	20/22 (90.90%)	10/27 (37.04%)	< *0.001* (*0.001*)	0.059
*PASD1*	17/22 (77.28%)	11/27 (40.74%)	0.019 (0.061)	0.202
*SCP1*	0/22 (0%)	0/27 (0%)	–	–
*SEMG1*	3/22 (13.63%)	0/27 (0%)	–	0
*SLLP1*	13/22 (59.10%)	8/27 (29.63%)	0.048 (0.113)	0.292
*SPANXA1*	2/22 (9.09%)	0/27 (0%)	–	0
*SSX1*	10/22 (45.45%)	14/27 (51.85%)	0.776 (0.873)	1.292
*PRAME*	22/22 (100%)	14/27 (51.85%)	< *0.001* (*0.001*)	0

CTA expression levels were standardized with log1p function with logarithm’s base equals

Statistically significant values are in italic

We found gene coexpression in the melanoma cells. Correlated expression was observed for levels of *GAGE* and *SLLP1* (rho = 0.588, adj. p = 0.019) on the one hand and for *SLLP1* and *SSX1* (rho = 0.582, adj. p = 0.020) on another.

We also found gene coexpression in STBS cells: correlation between *GAGE* and *NY*-*ESO1* (rho = 0.483, adj. p = 0.038), *MAGEA1* (rho = 0.498, adj. p = 0.035), *PASD1* (rho = 0.560, adj. p = 0.014); correlation between *NY*-*ESO1* and *MAGEA1* (rho = 0.556, adj. p = 0.014), *PRAME* (rho = 0.483, adj. p = 0.038); correlation between *MAGEA1* and *SLLP1* (rho = 0.488, adj. p = 0.038), *PRAME* (rho = 0.689, adj. p < 0.001).

**Fig. 2:**
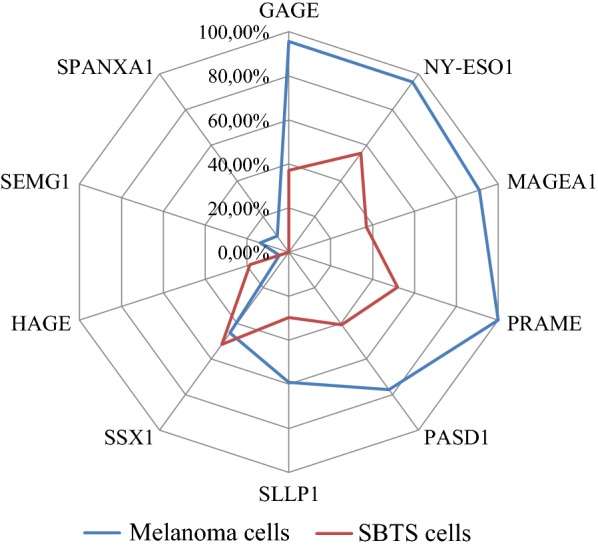
Incidence of CTA expression by cutaneous melanoma cells and soft tissue and bone sarcomas cells. We consider positive expression in the case of expression level above 0

Levels of expression of *GAGE*, *NY*-*ESO1*, *MAGEA1*, *PASD1*, *PRAME* in melanoma cell lines were significantly higher than in STBS group (adj. p < 0.05) (Table 2). Incidence of *GAGE*, *NY*-*ESO1*, *MAGEA1*, *PRAME* expression above 0 was significantly different (adj. p < 0.05) in melanoma and STBS groups (Fig. 2) when assessed separately. Meanwhile, profiles of expression of *HAGE*, *PASD1*, *SEMG1*, *SLLP1*, *SPANXA1*, *SSX1* weren’t significantly different in STBS and melanoma cell cultures at the complex assessment (Fig. 2).

**Fig. 3:**
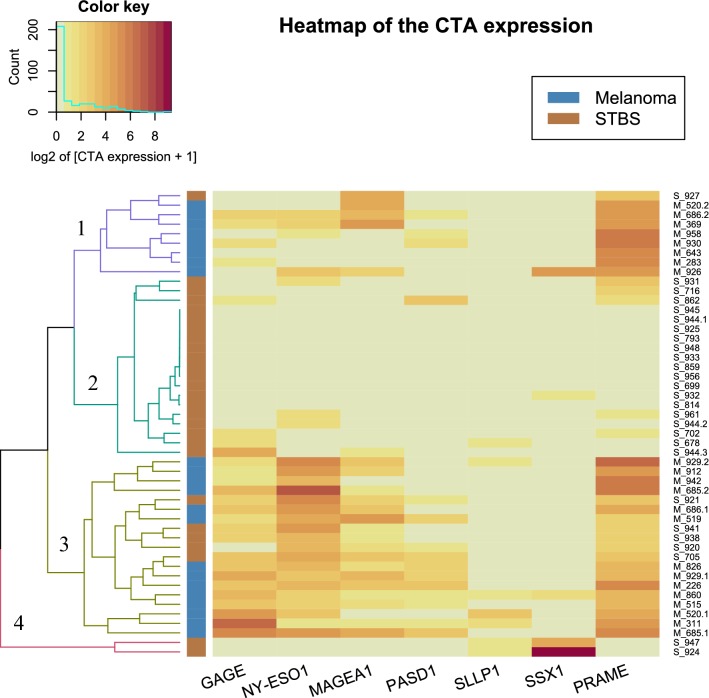
Results of the cluster analysis of the expression of the CTA cells, melanomas, and sarcomas. The intensity of expression is indicated as a log2 of expression of the target gene in relation to the reference gene expression plus one. Melanoma and STBS cultures are marked with blue and orange indicators, respectively

We conducted a complete-linkage hierarchical cluster analysis with Euclidean distance on the expression data. We applied R’s “NbClust” library for determining the appropriate number of clusters that performs computing 28 different indices and 2 graphical methods for that purpose. The final decision was made upon majority rule (see Additional files 1, 2, 3 and 4). The study revealed four distinct clusters of CTA expression (Fig. 3). In the first cluster, there were eight melanoma cell cultures and one STBS cell culture with low expression of *GAGE*, *NY*-*ESO1*, and *PASD1*, medium expression of *MAGEA1* in half of the cultures, and high expression of *PRAME.* The second cluster contained only STBS cells with an almost complete absence of CTA expression. In the third cluster, we got 14 melanoma cell cultures and 5 STBS cell cultures. The expression pattern is very similar to that in cluster №1, but with the higher expression in *GAGE, NY*-*ESO1, MAGEA1*, and *PASD1.* The last cluster contained only two STBS cell cultures with low expression of *SSLP1*, the highest expression of *SSX1*, and almost zero expression of the remaining CTA.

Generally, we have found three patterns of CTA expression: high expression of multiple CTA with some dominants of expression (#1, and #3), clusters with one hyper expressed CTA (#2) and cluster with no expression at all (#4).

### Interaction of antigen-specific cytotoxic T-lymphocytes with STBS cells correlates with CTA expression

Real-time monitoring of melanoma cells/STBS cells and T-lymphocytes activation was performed using the xCELLigence system.

**Fig. 4:**
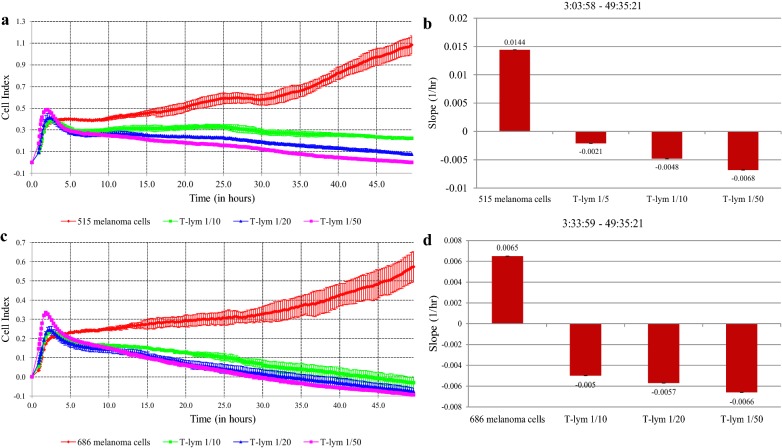
Interaction of specifically activated cytotoxic T-lymphocytes and melanoma cells: registration of the cellular index in time and the rate of culture growth under the influence of different amounts of T-lymphocytes. **a**, **b** Melanoma cells culture # 515; **c**, **d** melanoma cells culture # 686

We used the xCELLigence technology to monitor target cell killing in real-time. This method measures cell growth and proliferation through electrical impedance measurements. In the study, the electrical impedance was measured every 15 min after 1 h of observation, for 48–50 h. Cytotoxicity of T-cells has been assessed by a relative intensity of target cell growth in/without the presence of effector cells. In the first part of the experiment, changes in the level of melanoma cell proliferation during their co-cultivation with CTLs testified to the effect of CTLs on melanoma cells. We evaluated the efficacy of cytotoxic T-lymphocytes in skin melanoma cell lines 515 and 686, which were used to activate dendritic cells (Fig. 4a–d).

We have found the dependence of the cytotoxic effect of T-lymphocytes from their number, the maximum efficiency of the exposure recorded at a ratio of target/effector concentrations 1/50. The slope values of tumor cells proliferation rates were negative in the presence of T-lymphocytes, indicating inhibition of tumor cell culture growth, in contrast to the control culture without exposure.

**Fig. 5:**
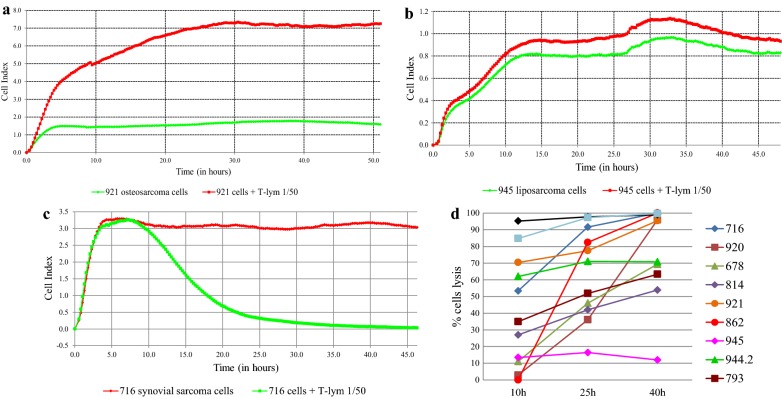
Interaction specifically activated cytotoxic T-lymphocytes and STBS cells: registration of the cellular index in time and the growth rate of the culture. **a** Osteogenic sarcoma 921; **b** liposarcoma 945; **c** synovial sarcoma 716; **d** dynamics of cell lysis in the process of interaction of T-lymphocytes and sarcoma cells

In the second part of the study, we used the most effective concentration of T-lymphocytes 1/50, and STBS cell lines expressing different amounts of the CTA (Fig. 5a–c) as target cells.

The minimal killing was 12% for culture 945, in the cells of which the genes of the studied CTA were not expressed. In four cases out of 11, cell killing was close to 100% (Fig. 5d). The efficacy of action on sarcoma cells, determined by CI change, correlated with the presence of expression of the gene *PRAME* (rho = 0.713, adj. p = 0.045) (Table 3).

**Table 3:** Correlation between the efficacy of action on sarcoma cells and the presence of expression in the genes (n = 11)

% lysis	Gene						
	*GAGE*	*NYESO1*	*MAGEA1*	*PASD1*	*SLLP1*	*SSX1*	*PRAME*
rho	0.239	− 0.067	0.116	0.415	0.032	− 0.248	0.713
p	0.479	0.845	0.734	0.205	0.926	0.461	0.014
adj. p	0.671	0.887	0.865	0.357	0.941	0.671	0.045

Interestingly enough, the clustering data corresponded to the efficiency of lysis, that is, the death of STBS cells in a cluster with a minimum level of CTA expression was less than 70% by 40 h of observation, while for cultures with a high level of expression this parameter was close to 100% within a day from the beginning of the experiment. Due to a limited number of cell lines tested, it is challenging to discover the best CTA expression profile as a predictor of immune therapy response on this data.

## Discussion

CTA are immunogenic for cancer patients. CTA exhibited highly tissue-restricted expression and are considered promising targets for cancer immunotherapy. We conducted a comparative study of gene expression of common classes of CTA cells with 22 melanoma and 27 STBS extracted from tumors of patients and long-term cultured in vitro. These cell cultures originated from metastatic disease and were extremely heterogeneous in morphological and growth characteristics. In our study, melanoma cells were characterized by a high level of CTA gene expression. The minimum number of active genes in melanoma cells was 4 out of 11, the maximum—8/11. The expression of the CTA was detected in 92.5% for STBS (25/27 samples). Thus, STBS may be characterized by a high incidence of CTA expression also. Scanlan et al. [33] proposed the classification of tumors according to the degree of severity expression of CTA. Tumors with high expression of the CTA are non-small cell lung cancer, and melanoma, with 11/20 (55%), 17/33 (51%) and 17/32 (53%) of the CTA transcripts examined by RT-PCR detected in 20% or more of the specimens examined, respectively. Breast and prostate cancer can be considered moderate CTA gene expressers, with 12/32 (37%) and 6/20 (30%) CTA transcripts having an expression frequency > 20%, respectively. Renal and colon cancer are low CT gene expressers, with only 3/33 (9%) and 4/25 (16%) CT transcripts having an expression frequency > 20%, respectively.

We have established that the universal antigens for this set of cell lines: GAGE, NY-ESO1, MAGEA1, PRAME, PASD1, and SSX1, since genes encoding them expressed more often than others. Different studies have noted a high frequency of NY-ESO1 expression in synovial sarcomas in particular. According to Jungbluth et al. [34], 20 out of 25 SS tumors were expressed NY-ESO1 by immunohistochemistry. The potential diagnostic role of NY-ESO1 expression was based on the detection of a high incidence of this antigen expression in synovial sarcomas, in comparison with other tumors of mesenchymal origin [35].

This data was the basis of further development of the vaccine based on NY-ESO1-activated DCs by transduction with a lentiviral vector. Such a vaccine has demonstrated high immunological efficiency in patients with refractory synovial sarcoma [36]. Accordingly, in the study by Jura et al. [23], immunohistochemically, NY-ESO1, PRAME, MAGEA4, and MAGEA1 were positive in 66 (61%), 93 (86%), 89 (82%), and 16 (15%) of 108 SSs, respectively, and 104 (96%) of 108 SSs showed the immunohistochemical expression of at least 1 of NYESO1, PRAME, and MAGEA4. Moreover, the high expression of at least one of these three antigens was observed in 83% of the SSs. High expression of NYESO1 and MAGEA4 significantly correlated with the presence of necrosis and advanced clinical stage [37]. In our study, myxofibrosarcoma (8/11), osteosarcoma (7/11), dermatofibrosarcoma (7/11), and synovial sarcoma (6/11) cells demonstrated the greatest expression of CTA genes. The activity of *MAGEA1* (25% 1/4), *NYESO1* (50% 2/4), and *PRAME* (75% 3/4) genes were also found in the cells of four samples of synovial sarcomas. Osteosarcoma was characterized by the presence of *GAGE* (40% 2/5), *MAGEA1* (60% 3/5), *PRAME* (40% 2/5), *NY*-*ESO1* (40% 2/5), *PASD1* (60% 3/5), *SLLP1* (60% 3/5), *SSX1* (80% 4/5).

Other studies reported *MAGEA* and *PRAME* expression in osteosarcoma [38]. They proposed to use *MAGEA* expression as a predictive biomarker for metastases (relative risk 2.79 (95% confidence interval 1.12–6.93; p = 0.028) for lung metastases in *MAGEA*-positive patients. Five-year survival rates for patients with and without *MAGEA* expression were 39.6% ± 8.4% and 80% ± 8.9% (M ± σ), respectively (log-rank test; p = 0.01) [38]. *PRAME* siRNA knockdown significantly suppressed proliferation, induced cell cycle stop in the G1 phase, reduced the efficiency of colony formation of cultured cells by osteosarcoma [37]. *PRAME* knockdown significantly suppressed the proliferation, colony formation, and G1 cell cycle arrest in osteosarcoma cells [37].

Hemminger et al. [39] have recorded a high homogeneous expression of the PRAME gene in the samples of myxoid and round cell liposarcoma. In our study, 2/4 liposarcoma samples were positive for the expression of this gene but characterized by a low grade of expression concerning the reference gene. Such a contradictory nature of the results obtained by different scientific groups can be explained by the high degree of heterogeneity of STBS in the expression of CTA gene activity. It is highly likely that this expression is individual and depends on many factors. Future research is mandatory to reveal them. According to Salmaninejad et al. [40], heterogeneity of expression levels of CTA genes was associated with DNA methylation. According to data obtained by Woloszynska-Read et al. [41] in the case of *NY*-*ESO1*, DNA methylation status was associated with both inter-tumor and intratumor heterogeneity of *NY*-*ESO1* expression in epithelial ovarian cancer. An exciting feature was observed in the expression of CTA in a number of studies: the complete absence of expression of all studied genes in some samples or the presence of multiple expression. Sahin et al. [42] showed 26% of melanoma cases with no CTA expression, while 52% of the tumor samples revealed expression of at least three CTAs.

Yao et al. [43] reported melanoma and lung cancer as tumors with the highest CTA expression, while kidney cancer and glioblastoma expressed CTA poorly. It can be assumed that the co-expression of CTA genes is associated with their role in the processes of invasion and metastasis. Molania et al. [44] has found that the co-examination of *PAGE4*, *SCP1*, and *SPANXA/D* may serve as a prognostic marker for the formation of metastases of colorectal cancer in the liver. Maine et al. [45] had demonstrated that the CTA genes *SPANXA/C/D* and *CTAG2* consistently induced in breast cancer cells and regulate distinct features of invasive behavior. There was a positive correlation between the source of the cell line (primary tumor or metastasis) and the expression of *NY*-*ESO1* and *PASD1* genes. So we can propose that their expression is associated with the metastatic potential of tumor cells.

On the other hand, we have shown three main types of CTA expression. Given the limited number of tested antigens, cases with no CTA expression could be those with hyperexpression of one CTA that was not selected in the panel. This hypothesis should be tested in further clinical trials. Several coexpressing CTA could also be of clinical significance. It is known that mutation load and the number of neoantigens are essential for immunotherapy efficacy, while tumors with one driver mutation usually have a lower mutational burden and lower dependency from the immune system.

Despite the more pronounced expression of the CTA in melanoma cells, comparison of the qualitative expression profiles in melanoma and STBS confirms similar patterns of their expression in these tumors. These patterns give the opportunity to use melanoma cells as the source of rich CTA lysate for the treatment of STBS patients. Melanoma cells have an advantage in the low complexity of their transfer to culture, more intense proliferation, and the ability to increase a large mass of identical cell material in a short time. These advantages can meaningfully improve the practical usage of DC technology for cancer treatment. We have tested this proposal in the in vitro system by the interaction of specifically activated cytotoxic CD8^+^ T lymphocytes and STBS cells. This was done with 11 variants of sarcoma cell lines using the xCELLigence cell analyzer system. The efficacy of target cell lysis was individual for each cell line and varied over time, but correlated with the presence of expression of the *PRAME*. Pollack et al. [46] showed the possibility of using the NY-ESO1 antigen for specific activation of CTLs against myxoid/round cell liposarcoma cells for which its homogeneous expression was detected. Our data does not support this. Yet, one should consider a highly heterogeneous expression of the most types of CTA in ours. So, we can propose that the expression profile of each tumor sample can play a role in determining lysis efficacy. The exact contribution of different CTA in this process should be further investigated. Antitumor vaccines are well known promising modality of immunotherapy, that had caused a considerable disappointment due to several negative trials, including large studies with CancerVax [47] and DERMA trial [48]. New sophisticated vaccination strategies, called “second-generation vaccine” DC technologies have entirely different perspectives in the patient management and are actively studied now [2]. Antigen source and quality are one of the milestones of these technologies that has a crucial effect on therapeutic efficacy. Our data shows that patterns of CTA expression could be used for effective targeting of different histological types of tumors. Clinical data from our center support this evidence [49]. Advances from both rich allergenic material and autologous DC could be used for developing effective immunotherapeutic decisions.

## Conclusion

A similar antigenic profile of the skin melanoma cell lines and soft tissue and bone sarcomas allows using selected skin melanoma cells for the preparation of lysates used for the production of dendritic cell vaccines. The role of CTA expression patterns should be tested in prospective clinical trials.

## Supplementary information

**Additional file 1: Figure S1.** Scatter matrix of the gene co-expression with histograms and results of correlation analysis for melanoma cell cultures.

**Additional file 2: Figure S2.** Scatter matrix of the gene co-expression with histograms and results of correlation analysis for STBS cell cultures.

**Additional file 3: Figure S3.** Graphical methods for determining the relevant number of clusters. R’s “NbClust” output for the relevant number of clusters. Among all indices: 3 proposed 2 as the best number of clusters; 4 proposed 3 as the best number of clusters; *9 proposed 4 as the best number of clusters*; 1 proposed 8 as the best number of clusters; 5 proposed 11 as the best number of clusters; 1 proposed 13 as the best number of clusters; 5 proposed 15 as the best number of clusters. Both graphical methods (Hubert and D indexes) also proposed 4 as the best number of clusters.

**Additional file 4.** Study dataset with clusters highlighted by colors.

## Data Availability

The datasets used and analyzed during the current study are available from the corresponding author on reasonable request.
