# Supplementary figures and images for: Cancer/testis antigens expression during cultivation of melanoma and soft tissue sarcoma cells
Source: Clin Sarcoma Res. 2020 Feb 4;10:3. doi: 10.1186/s13569-020-0125-2 (PMC6998350; doi:10.1186/s13569-020-0125-2)

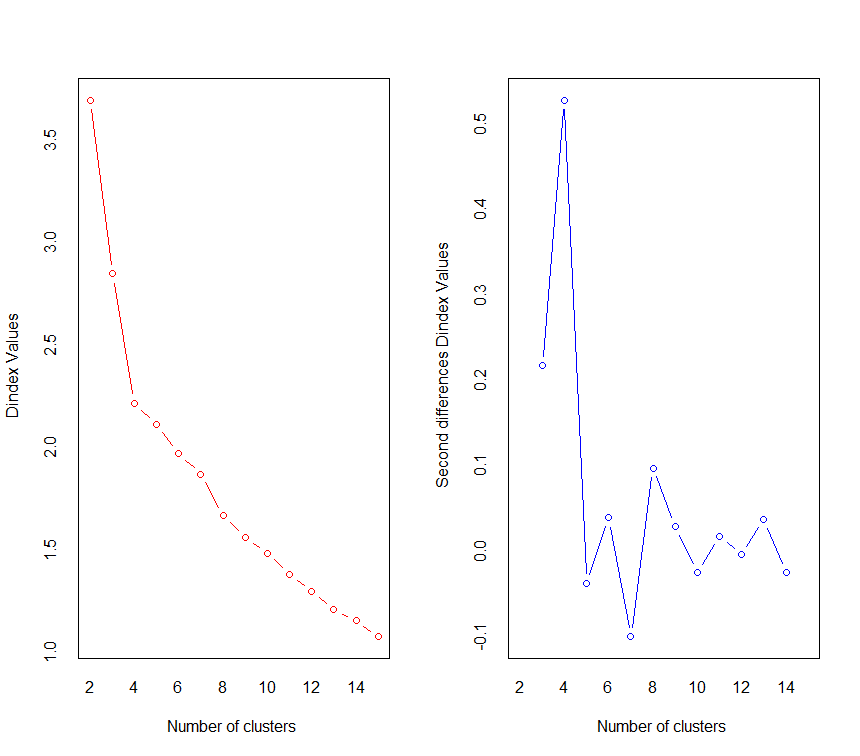

Supplement: Supplementary file 3 — Additional file 3: Figure S3. Graphical methods for determining the relevant number of clusters. R’s “NbClust” output for the relevant number of clusters. Among all indices: 3 proposed 2 as the best number of clusters; 4 proposed 3 as the best number of clusters; 9 proposed 4 as the best number of clusters; 1 proposed 8 as the best number of clusters; 5 proposed 11 as the best number of clusters; 1 proposed 13 as the best number of clusters; 5 proposed 15 as the best number of clusters. Both graphical methods (Hubert and D indexes) also proposed 4 as the best number of clusters. [file 13569_2020_125_MOESM3_ESM.png]
